# *KTN1* Variants Underlying Putamen Gray Matter Volumes and Parkinson’s Disease

**DOI:** 10.3389/fnins.2020.00651

**Published:** 2020-06-23

**Authors:** Qiao Mao, Xiaoping Wang, Bin Chen, Longhua Fan, Shuhong Wang, Yong Zhang, Xiandong Lin, Yuping Cao, Yun-Cheng Wu, Jiawu Ji, Jianying Xu, Jianming Zheng, Huihao Zhang, Chengchou Zheng, Wenzhong Chen, Wenhong Cheng, Xingqun Luo, Kesheng Wang, Lingjun Zuo, Longli Kang, Chiang-Shan R. Li, Xingguang Luo

**Affiliations:** ^1^Department of Psychosomatic Medicine, People’s Hospital of Deyang, Deyang, China; ^2^Department of Neurology, Shanghai Tongren Hospital, Shanghai Jiao Tong University, Shanghai, China; ^3^Department of Cardiovascular Medicine, Fujian Provincial Hospital, Fuzhou, China; ^4^Qingpu Branch, Department of Vascular Surgery, Zhongshan Hospital, Fudan University, Shanghai, China; ^5^Tianjin Mental Health Center, Tianjin, China; ^6^Laboratory of Radiation Oncology and Radiobiology, Fujian Provincial Cancer Hospital, Teaching Hospital of Fujian Medical University, Fuzhou, China; ^7^Department of Psychiatry, Second Xiangya Hospital, Central South University, Changsha, China; ^8^Department of Neurology, Shanghai General Hospital, Shanghai Jiao Tong University School of Medicine, Shanghai, China; ^9^Department of Psychiatry, Fuzhou Neuropsychiatric Hospital, Fujian Medical University, Fuzhou, China; ^10^Zhuhai Municipal Maternal and Children’s Health Hospital, Zhuhai, China; ^11^Huashan Hospital, Fudan University School of Medicine, Shanghai, China; ^12^The First Affiliated Hospital, Fujian Medical University, Fuzhou, China; ^13^Minqing Psychiatric Hospital, Minqing, China; ^14^Department of Psychiatry, Shanghai Mental Health Center, Shanghai, China; ^15^Department of Clinical Medicine, College of Integrated Traditional Chinese and Western Medicine, Fujian University of Traditional Chinese Medicine, Fuzhou, China; ^16^Department of Family and Community Health, School of Nursing, Health Sciences Center, West Virginia University, Morgantown, WV, United States; ^17^Department of Psychiatry, Yale University School of Medicine, New Haven, CT, United States; ^18^Key Laboratory for Molecular Genetic Mechanisms and Intervention Research on High Altitude Diseases of Tibet Autonomous Region, Xizang Minzu University School of Medicine, Xiangyang, China; ^19^Biological Psychiatry Research Center, Beijing Huilongguan Hospital, Beijing, China

**Keywords:** Parkinson’s disease, *KTN1*, putamen, substantia nigra, gray matter volume, mRNA expression

## Abstract

**Background:**

Selective loss of dopaminergic neurons and diminished putamen gray matter volume (GMV) represents a central feature of Parkinson’s disease (PD). Recent studies have reported specific effects of kinectin 1 gene (*KTN1*) variants on the putamen GMV.

**Objective:**

To examine the relationship of *KTN1* variants, *KTN1* mRNA expression in the putamen and substantia nigra pars compacta (SNc), putamen GMV, and PD.

**Methods:**

We examined the associations between PD and a total of 1847 imputed *KTN1* single nucleotide polymorphisms (SNPs) in one discovery sample [2,000 subjects with PD vs. 1,986 healthy controls (HC)], and confirmed the nominally significant associations (*p* < 0.05) in two replication samples (900 PD vs. 867 HC, and 940 PD vs. 801 HC, respectively). The regulatory effects of risk variants on the *KTN1* mRNA expression in putamen and SNc and the putamen GMV were tested. We also quantified the expression levels of *KTN1* mRNA in the putamen and/or SNc for comparison between PD and HC in five independent cohorts.

**Results:**

Six replicable and two non-replicable *KTN1*-PD associations were identified (0.009 ≤ *p* ≤ 0.049). The major alleles of five SNPs, including rs12880292, rs8017172, rs17253792, rs945270, and rs4144657, significantly increased risk for PD (0.020 ≤ *p* ≤ 0.049) and putamen GMVs (19.08 ≤ β ≤ 60.38; 2.82 ≤ Z ≤ 15.03; 5.0 × 10^–51^ ≤ *p* ≤ 0.018). The risk alleles of five SNPs, including rs8017172, rs17253792, rs945270, rs4144657, and rs1188184 also significantly increased the *KTN1* mRNA expression in the putamen or SNc (0.021 ≤ *p* ≤ 0.046). The *KTN1* mRNA was abundant in the putamen and/or SNc across five independent cohorts and differentially expressed in the SNc between PD and HC in one cohort (*p* = 0.047).

**Conclusion:**

There was a consistent, significant, replicable, and robust positive relationship among the *KTN1* variants, PD risk, *KTN1* mRNA expression in putamen, and putamen volumes, and a modest relation between PD risk and *KTN1* mRNA expression in SNc, suggesting that *KTN1* may play a functional role in the development of PD.

## Introduction

The nigrostriatal dopaminergic pathway connects the substantia nigra pars compacta (SNc) with the dorsal striatum, forms part of the extrapyramidal system, and plays a central role in motor control (Tepper and Lee, 2007; Tritsch et al., 2012). Selective loss of dopaminergic neurons in the SNc represents a cardinal pathological feature of Parkinson’s disease (PD), and consequent dopamine depletion in the striatum results in marked motor deficits, including tremors, rigidity, hypokinesia and postural imbalance (Salkov and Khudoerkov, 2018).

Consistent with the loss of dopaminergic neurons, imaging studies have demonstrated altered nigrostriatal functions in PD (Deumens et al., 2002). For example, individuals with PD, as compared to healthy controls, showed decreases in the amplitude of low-frequency fluctuation of blood oxygenation-level dependent signals in the putamen (Wang et al., 2018). Many studies described altered functional connectivity of the putamen in PD (Wang et al., 2017; Liu et al., 2018; Manes et al., 2018), including decreases in connectivity with orbitofrontal gyrus and cerebellum (Wang et al., 2017), and increases in connectivity with the supplementary motor area (Yu et al., 2013) and caudate (Wang et al., 2017). In positron emission tomography imaging, expression of the serotonin transporter (Kish et al., 2008) appeared to decrease, and the size of dopamine transporter/α-synuclein complexes (components of the Lewy bodies) increased in the putamen in PD (Longhena et al., 2018).

Furthermore, in the SNc, the expression levels of calbindin (Blesa and Vila, 2019), gangliosides GM1, GD1a, GD1b, and GT1b, ganglioside biosynthetic genes *B3GALT4* and *ST3GAL2* (Schneider, 2018), and ghrelin receptors (Suda et al., 2018) were absent or significantly decreased in PD. The expression levels of glycoprotein GPNMB (Moloney et al., 2018), SNc free water (Guttuso et al., 2018), and iron accumulation (Barbosa et al., 2015; An et al., 2018) were significantly elevated; and mitochondrial function was altered (Reeve et al., 2018) in PD. These findings support altered molecular cascades and physiological processes in the nigrostriatal pathways in PD. Finally, many treatments of PD target the putamen and/or SNc, including L-DOPA as the first-line medication. L-DOPA restores dopaminergic signaling and improves motor control including response inhibition by enhancing striatal activation in early-stage Parkinson’s disease (Manza et al., 2018). High-frequency deep brain stimulation of the putamen (Montgomery et al., 2011), low-frequency deep brain stimulation of the SN pars reticulata (Valldeoriola, 2019; Weiss et al., 2019), and crocin (Haeri et al., 2019) for treatment-refractory patients have also demonstrated efficacy in the treatment of PD. On the basis of these findings, we focused on the putamen and SNc in the current study.

The nigrostriatal pathway may be genetically controlled. A genetic marker at 3′-UTR of kinectin 1 gene (*KTN1*), i.e., rs945270, demonstrated the genome-wide strongest (*p* = 1.1 × 10^–33^), replicable, and specific effects on the putamen gray matter volume (GMV) in subjects without neurodegenerative or neuropsychiatric disorders (Hibar et al., 2015; Xu et al., 2017). The other three markers at *KTN1*, i.e., rs2181743 (5′-UTR), rs8017172 (3′-UTR), and rs17253792 (3′-UTR), significantly increased the putamen GMVs too [*p* = 4.0 × 10^–8^ (6.7 × 10^–34^ to 3.0 × 10^–14^) and 3.2 × 10^–7^, respectively] (Chen et al., 2017; Satizabal et al., 2019). Among them, rs8017172 has been reported to significantly *cis*-regulate the methylation of CpG islands in the putamen (*p* = 4.4 × 10^–6^) (Satizabal et al., 2019). Except for these four variants reported to regulate the putamen GMVs, no other *KTN1* variants have been reported to influence the putamen and SNc GMVs. Importantly, these four variants are located in the same haplotype block. These findings together suggest a relationship among *KTN1* variants, *KTN1* expression in putamen, and putamen GMV.

**FIGURE 1:**
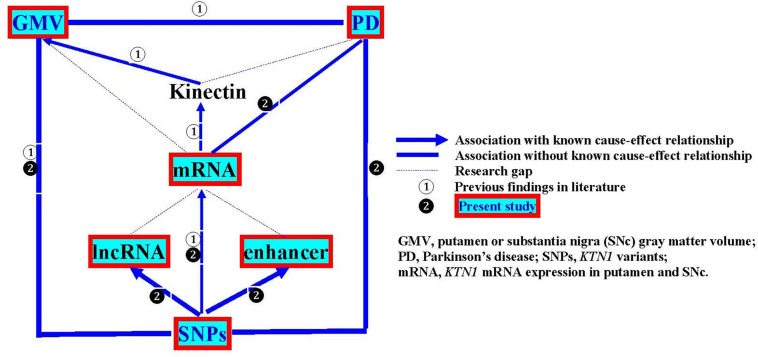
Regulatory pathway from genotypes to phenotypes.

In the present study, we aimed to examine the *KTN1* variants as a genetic risk factor for PD, the roles of the *KTN1* variants in regulating mRNA expression in the putamen and SNc and putamen GMV, and whether *KTN1* mRNA may be differentially expressed in the putamen and SNc between PD and controls. The overall design of this study is illustrated in Figure 1.

## Materials and Methods

### Subjects

We examined three independent population-based Caucasian samples: “PD_ENV” (dbGaP access number: phs000196.v3.p1), “phg000022” (phs000126.v2.p1), and “lng_coriell_pd” (phs001172.v1.p2). The first sample served as the discovery sample and the other two served as the replication samples. The discovery sample included 2,000 subjects with PD (1,346 males and 654 females) and 1,986 healthy subjects (769 males and 1,217 females). The first replication sample, i.e., “phg000022,” included 900 subjects with PD (537 males and 363 females) and 867 healthy subjects (363 males and 521 females). The second replication sample, i.e., “lng_coriell_pd,” included 940 subjects with PD (560 males and 380 females) and 801 healthy subjects (336 males and 465 females).

All cases met the UK Parkinson’s Disease Society Brain Bank clinical diagnostic criteria for PD (Gibb and Lees, 1988), and were excluded if their initial PD diagnosis changed during the ∼12 years of follow-up, or they had other neurologic or neurodegenerative conditions, or psychotic, mood and substance use disorders. All controls were free from PD and other neuropsychiatric and neurodegenerative diseases. Diagnoses of these subjects have been confirmed by physician interviews, questionnaires, hospital medical records, as well as pathology, radiology and neuropsychology reports. All subjects are Caucasians. The demographic data of these three samples have been described in detail before (Nichols et al., 2007; Simon-Sanchez et al., 2009; Hamza et al., 2010). All study procedures were reviewed and approved by the Human Investigation Committee of all institutions.

### Imputation

The discovery sample was genotyped on Illumina HumanOmni1_Quad_v1-0_B microarray platform. The first replication sample “phg000022” was genotyped on Illumina HumanCNV370v1 microarray platform, and the second replication sample “lng_coriell_pd” was genotyped by Whole Exome Sequencing using Illumina TruSeq system. To make the genetic marker sets consistent across different samples, we imputed the entire *KTN1* region (Chr14:54995382-55550419) by the program IMPUTE2 (Howie et al., 2009). The imputed data were stringently “cleaned up” prior to association analysis (Zuo et al., 2012; Luo et al., 2020a).

### Gene-Disease Association Analysis

SNP-PD associations were analyzed using logistic regression models as implemented in the program PLINK (Purcell et al., 2007), in which the diagnosis served as dependent variable, alleles as independent variables, and sex and age as covariates. The associations in the discovery sample were analyzed first. A *p* < 0.05 indicates a nominally significant association. These nominal associations were further explored in the two replication samples. A SNP-PD association with *p* < 0.05 in both discovery and replication samples was taken as a replicable association.

### Bioinformatic Analyses

A series of bioinformatic analyses, including FuncPred (Xu and Taylor, 2009) and VE!P (McLaren et al., 2010) and the UCSC Genome Browser, were conducted to predict the potential biological functions of the risk SNPs, to explore the relationship of the risk SNPs with DNA or RNA transposons, long non-coding RNAs (lncRNAs), transcription factor binding sites (TFBS), and enhancers. Finally, we reviewed the literature for the regulatory effects of these risk SNPs on the GMVs of putamen and SNc.

### Associations of PD-Risk Alleles With *KTN1* mRNA Expression in Putamen and SNc, and With Putamen GMV

After the risk *KTN1* alleles for PD were identified from the afore-described gene-disease association analyses, the potential regulatory effects of the risk alleles on the *KTN1* mRNA expression in human postmortem putamen and SNc in a UK European cohort (*n* = 129) (BRAINEAC dataset) (Ramasamy et al., 2014) and a European-American cohort (*n* = 170) (GTEx dataset) (Gtex Consortium, 2013) were analyzed using *cis*-acting expression quantitative trait locus (*cis-*eQTL) analysis.

The potential regulatory effects of these risk alleles on the putamen GMV were analyzed in two European postmortem putamen samples (*n* = 13,145 and 37,571, respectively) [Enhancing Neuro Imaging Genetics through Meta-Analysis (ENIGMA2) consortium – GWAS Meta-Analysis of Subcortical Volumes]^1^ (Hibar et al., 2015; Satizabal et al., 2019) using multiple linear regression analysis. These subjects were free of neurodegenerative and neuropsychiatric disorders. The β- and *Z*-values, measures of effect sizes, and the p values, a measure of statistical significance, from the regression models were calculated.

### *KTN1* mRNA Expression in Putamen and SNc

The *KTN1* mRNA expression in the putamen and SNc was examined in the postmortem brains of five independent cohorts. Cohorts 1–5 comprised 129 putamen and 101 SNc tissues [UK Brain Expression Consortium (UKBEC)] (Ramasamy et al., 2014), 124 putamen and 88 SNc tissues [The Genotype-Tissue Expression (GTEx) project (Gtex Consortium, 2013)], 15 putamen and 15 SNc tissues (BioGPS) (Zhang et al., 2005), 9 SNc tissues (BioGPS) (Papapetropoulos et al., 2006; Lesnick et al., 2007), and 14 SNc tissues (BioGPS) (Zheng et al., 2010) extracted from the subjects without neuropsychiatric and neurodegenerative disorders, respectively. Cohorts 3–5 also comprised 15 putamen and 11 SNc tissues (BioGPS) (Zhang et al., 2005), 16 SNc tissues (BioGPS) (Papapetropoulos et al., 2006; Lesnick et al., 2007), and 14 SNc tissues (BioGPS) (Zheng et al., 2010) extracted from the subjects with PD, respectively. The subjects were either UK Europeans (Cohort 1) or European-Americans (Cohorts 2–5). mRNA expression in Cohorts 1, 3, 4, and 5 was examined using Affymetrix Human ST 1.0 exon arrays or Affymetrix Human U133A GeneChips (validated by qPCR). The expression levels with normalized intensity >36, i.e., log_2_(normalized intensity) >5.17, were taken as “expressed.” mRNA expression in Cohort 2 was examined using RNA-Seq (validated by qPCR). The expression levels with RPKM values >1 were taken as “expressed” (Luo et al., 2020b).

As the putamen volume decreases with age (McDonald et al., 1991; Greven et al., 2015; Halkur Shankar et al., 2017), the expression levels of *KTN1* mRNA in the putamen and/or SNc in Cohorts 3, 4, and 5 were compared between PD and healthy subjects using analysis of covariance (ANCOVA) with age as a covariate. A *p* < 0.05 indicates significantly different expressions.

## Results

### Replicable Associations Between *KTN1* SNPs and PD Across Discovery and Replication Samples (Table 1)

**FIGURE 2:**
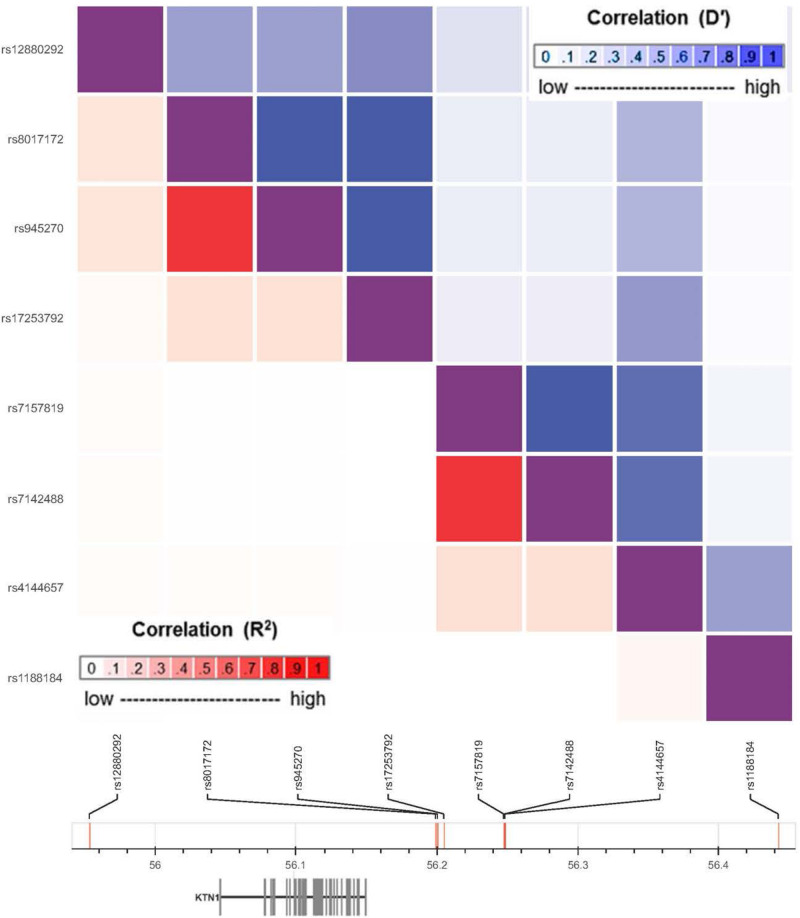
Haplotype blocks of risk *KTN1* variants for Parkinson’s disease.

A total of 1847 imputed *KTN1* SNPs were analyzed in the discovery sample (“PD_ENV”), including 142 SNPs nominally associated with PD (*p* < 0.05). Among them, six SNPs significantly associated with PD in the discovery sample were also associated with PD in at least one replication sample (“phg000022” or “lng_coriell_pd”), including four replicable associations between “PD_ENV” and “phg000022” samples (0.009 ≤ *p* ≤ 0.049) and two replicable associations between “PD_ENV” and “lng_coriell_pd” samples (0.020 ≤ *p* ≤ 0.045). These six replicable risk SNPs, together with the other two non-replicable GMV-associated variants (i.e., rs8017172 and rs945270) (Hibar et al., 2015; Satizabal et al., 2019), were located in four haplotype blocks (D’ > 0.8; Figure 2), including one in 5′-UTR and the others in 3′-UTR. The major alleles G of rs8017172, T of rs17253792, and C of rs945270 in the H2 block, the three that have been significantly associated with increases in putamen GMVs (*p* = 2.5 × 10^–24^, 3.2 × 10^–7^, and 1.1 × 10^–33^, respectively) (Hibar et al., 2015; Chen et al., 2017), were positively associated with PD in the discovery sample (*p* = 0.049 for rs17253792) and/or the replication sample “phg000022” (*p* = 0.021 for rs8017172, *p* = 0.043 for rs17253792, and *p* = 0.021 for rs945270, respectively).

**TABLE 1:** Bioinformatics of *KTN1* SNPs significantly associated with Parkinson’s disease in three independent European-American samples.

	**Location**	**Genomic**	**Frequency of**			***p*-values for associations with PD**				
	**(Haplotype**	**Position**	**Risk allele**			**Discovery**	**Replication 1**	**Replication 2**		
**SNP**	**Blocks, H)**	**at 14q**	**Allele**	**PD**	**Control**	**(*n* = 3,986)**	**(*n* = 2,082)**	**(*n* = 1,741)**	**Function class**	**Transposons (size; type)**
rs12880292	5′-UTR (H1)	55022617	G	0.693	0.672	0.045	–	0.020	–	Arthur1B (163bp; DNA)
rs8017172*	3′-UTR (H2)	55268801	G	0.609	0.570	–	0.021	–	GMV, eQTL	MER5A (150bp; DNA)
rs17253792*	3′-UTR (H2)	55274783	T	0.945	0.933	0.049	0.043	–	GMV, eQTL	–
rs945270*	3′-UTR (H2)	55270226	C	0.623	0.583	–	0.021	–	GMV, eQTL	–
rs7157819	3′-UTR (H3)	55317346	T	0.312	0.285	0.014	0.018	–	lincRNA	–
rs7142488	3′-UTR (H3)	55317763	C	0.317	0.289	0.009	0.014	–	lincRNA	–
rs4144657	3′-UTR (H3)	55318208	G	0.935	0.902	0.025	–	0.020	lincRNA	–
rs1188184	3′-UTR (H4)	55512165	A	0.339	0.295	0.043	0.014	–	enhancer	–

**Three independent samples: Discovery sample (“PD_ENV”), and replication samples 1 (“phg000022”) and 2 (“lng_coriell_pd”). PD, Parkinson’s disease; n, sample size of a total sample. lincRNA, located in a long interspersed ncRNA sequence; enhancer, located in an enhancer; transposons, located in transposons that include DNA transposons and RNA retrotransposons (SINE and LINE). ^∗^These three variants were previously reported to regulate KTN1 mRNA expression in putamen (eQTL) and the putamen gray matter volume (GMV), including two non-replicable variants, i.e., rs8017172 and rs945270.**

### The Risk *KTN1* SNPs May Be Biologically Functional (Table 1)

The eight risk variants were located in four haplotype blocks. Bioinformatic analysis showed that the variants within the same haplotype blocks were not only highly linked but also shared similar biological functions. All of the three variants in H3 were located in long intergenic non-coding RNAs (lincRNAs). The variant in H4, i.e., rs1188184, was located in an enhancer. Furthermore, two risk variants were, respectively, located on two transposons, including rs12880292 on the DNA transposon Arthur1B (163 bp), and rs8017172 on the DNA transposon MER5A (150 bp).

### The PD-Risk Alleles Potentially Increased the *KTN1* mRNA Expression in Putamen and SNc, and the Putamen GMV (Table 2)

The alleles with significantly higher frequencies in the PD groups than the controls were identified as the risk alleles for PD by the afore-mentioned association analyses. The risk alleles of rs8017172 and rs945270 increased the *KTN1* mRNA expression in putamen both in UK Europeans (*p* = 0.049 for both; BRAINEAC dataset) and European-Americans (*p* = 0.029 and 0.021, respectively, GTEx dataset). Two risk alleles of rs17253792 and rs1188184 increased mRNA expression in putamen in European-Americans too (*p* = 0.030 and 0.046, respectively). Additionally, the risk allele of rs4144657 increased mRNA expression in SNc in European-Americans (*p* = 0.045). Four risk alleles of rs8017172, rs17253792, rs945270 and rs4144657 were all major alleles (*f* > 0.5), consistent with previous reports (Gtex Consortium, 2013; Ramasamy et al., 2014).

**TABLE 2:** Associations between the PD-risk SNPs and both *KTN1* mRNA expression in putamen or substantia nigra and GMVs of putamen.

		**SNP-mRNA association**						**SNP-GMV association**			
		**UK Europeans**		**European-Americans**				**Europeans**			
		**BRAINEAC**		**GTEx**				**ENIGMA2 sample 1**		**ENIGMA2 sample 2**	
		**Putamen (*n* = 129)**		**Putamen (*n* = 170)**		**Substantia Nigra (*n* = 114)**		**Putamen (*n* = 13,145)**		**Putamen (*n* = 37,571)**	
						
**SNP**	**PD-risk allele**	**Effect size**	***p*-value**	**Effect size**	***p*-value**	**Effect size**	***p*-value**	**Effect size (β)**	***p*-value**	**Effect size (Z)**	***p*-value**
rs12880292	G		>0.05		>0.05		>0.05	+19.08	0.002	+ 3.80	1.5 × 10^–4^
rs8017172	G	+	0.049	+0.07	0.029		>0.05	+60.38	5.7 × 10^–24^	+15.02	5.5 × 10^–51^
rs17253792	T		>0.05	+0.37	0.030		>0.05	+52.82	1.9 × 10^–7^	+ 6.86	7.0 × 10^–12^
rs945270	C	+	0.049	+0.08	0.021		>0.05	+48.90	1.1 × 10^–33^	+15.03	5.0 × 10^–51^
rs7157819	T		>0.05		>0.05		>0.05		>0.05		>0.05
rs7142488	C		>0.05		>0.05		>0.05		>0.05		>0.05
rs4144657	G		>0.05		>0.05	+0.18	0.045	+28.38	0.018	+ 2.82	0.005
rs1188184	A		>0.05	+0.07	0.046		>0.05		>0.05		>0.05

**SNPs in this table correspond to Table 1. GMV, gray matter volume; BRAINEAC data, mRNA expression data in European brains from UK Brain Expression Consortium (UKBEC); GTEx data, mRNA expression data in European American brains from Genotype-Tissue Expression (GTEx) project; ENIGMA2 data, imaging genomic data for subcortical structure in European brains from Enhancing Neuro Imaging Genetics Through Meta-Analysis project.**

The risk alleles of five SNPs increased the GMVs of putamen in both ENIGMA2 European samples, which included rs12880292 in H1 block (β = 19.08, *p* = 0.002 in Sample 1; *Z* = 3.80, *p* = 1.5 × 10^–4^ in Sample 2) and rs4144657 in H3 block (β = 28.38, *p* = 0.018; *Z* = 2.82, *p* = 0.005) that modestly increased the putamen GMVs, and rs8017172 (β = 60.38, *p* = 5.7 × 10^–24^; *Z* = 15.02, *p* = 5.5 × 10^–51^), rs17253792 (β = 52.82, *p* = 1.9 × 10^–7^; *Z* = 6.86, *p* = 7.0 × 10^–12^) and rs945270 (β = 48.90, *p* = 1.1 × 10^–33^; *Z* = 15.03, *p* = 5.0 × 10^–51^) in H2 block that highly significantly increased the putamen GMVs. All of these five risk alleles were major alleles (*f* > 0.5). These SNP-GMV associations were consistent with previous reports (Hibar et al., 2015; Chen et al., 2017; Satizabal et al., 2019).

### The *KTN1* mRNA Was Significantly Expressed in the Putamen and/or SNc Across Five Independent Cohorts and Differentially Expressed in the SNc Between PD and Healthy Controls in One Cohort (Table 3)

In three independent cohorts, Cohorts 1, 2, and 3, the *KTN1* mRNA was abundantly expressed in the putamen. The expression levels in control subjects were 5.59 ± 0.36 [log_2_(normalized intensity)], 38.70 [Transcripts Per Kilobase Million (TPM)] and 9.79 ± 0.62 [log_2_(normalized intensity)], respectively. *KTN1* mRNA was also abundantly expressed in PD subjects in Cohort 3 (expression level = 9.86 ± 0.44), at a level higher than control subjects, although the difference was not statistically significant (*p* = 0.858).

**TABLE 3:** The *KTN1* mRNA expression in putamen or substantia nigra with/without Parkinson’s disease (PD) in five independent cohorts.

	**Cohort 1**		**Cohort 2**		**Cohort 3**		**Cohort 4**	**Cohort 5**
Populations	UK Europeans		European-Americans		European-Americans		European-Americans	European-Americans
Dataset names	BRAINEAC		GTEx		BioGPS		BioGPS	BioGPS
References	Ramasamy et al., 2014		Gtex Consortium, 2013		Zhang et al., 2005		Lesnick et al., 2007	Zheng et al., 2010
Experiment methods	Affymetrix Human ST 1.0 exon arrays		RNA-Seq		Affymetrix Human U133A GeneChip		Affymetrix Human U133+ GeneChip	Affymetrix Human U133A GeneChip
Measurement of expression	Log_2_(normalized intensity)		Transcripts Per Kilobase Million (TPM)		Log_2_(normalized intensity)		Log_2_(normalized intensity)	Log_2_(normalized intensity)
Expression threshold			1				5.17	5.17
**Normal subjects**								
Brain disorders	5.17	No	No		5.17	No	No	No
Ages at death (years)	59 ± 25 (16–102)		41 ± 14 (21–70)		71 ± 11 (54–94)		78 ± 13 (46–90)	77 ± 12 (52–88)
Tissue types	Putamen	Substantia Nigra	Putamen	Substantia Nigra	Putamen	Substantia Nigra	Substantia Nigra	Substantia Nigra
Sample sizes	129	101	124	88	15	15	9	14
Expression levels	5.59 ± 0.36	5.76 ± 0.35	38.7	55.7	9.79 ± 0.62	10.04 ± 0.75	10.76 ± 0.26	9.18 ± 0.49
**Patient subjects**								
Brain disorders					PD	PD	PD	PD
Ages at death (years)					77 ± 6 (67–89)	75 ± 6 (67–84)	75 ± 8 (60–88)	80 ± 6 (74–87)
Tissue types					Putamen	Substantia Nigra	Substantia Nigra	Substantia Nigra
Sample sizes					15	11	16	14
Expression levels					9.86 ± 0.44	10.03 ± 0.50	10.95 ± 0.19	9.14 ± 0.31
*p*-values for ANCOVA					0.858	0.909	0.047	0.814

**The data of putamen in Cohorts 1–3 were previously reported by Luo et al. (2020b).**

Across the five independent cohorts, the *KTN1* mRNA was abundantly expressed in the SNc too. The expression levels in control subjects were 5.76 ± 0.35 [log_2_(normalized intensity)], 55.70 [TPM], 10.04 ± 0.75 [log_2_(normalized intensity)], 10.76 ± 0.26 [log_2_(normalized intensity)], and 9.18 ± 0.49 [log_2_(normalized intensity)], for Cohorts 1–5, respectively. Furthermore, the expression levels in PD subjects in Cohorts 3, 4, and 5 were 10.03 ± 0.50, 10.95 ± 0.19, and 9.14 ± 0.31 [log_2_(normalized intensity)], respectively. The expression levels were significantly higher in PD than in control subjects in Cohort 4 (*p* = 0.047) but were not different for Cohorts 3 and 5 (*p*’s > 0.05).

## Discussion

We found six *KTN1* variants that were associated with PD across at least two independent samples, and two other GMV-associated variants that were associated with PD in one sample. Six of these risk variants may be biologically functional, regulating either the mRNA expression in putamen or SNc or the GMVs of putamen. *KTN1* mRNAs were expressed in the putamen and/or SNc across five independent cohorts, and were differentially expressed in the SNc between PD and controls in one cohort. Together, these results suggest that *KTN1* plays a functional role in the development of PD, supporting previous findings of associations between *KTN1* variants and PD (van Dijk et al., 2012; Nalls et al., 2014; Chang et al., 2017).

Two risk variants located in DNA transposons may control the transcription of *KTN1*, playing a decisive mutagenic role in degenerative pathologies (O’Donnell and Burns, 2010; Li et al., 2013; Sturm et al., 2017), most obviously in the brain (De Cecco et al., 2013; Van Meter et al., 2014). Three risk variants in H3 located in a lincRNA may regulate *KTN1* mRNA expression. One variant located in an enhancer may affect the transcription too. The potential biological functions of PD-risk SNPs, along with the abundant *KTN1* mRNA expression in the putamen and SNc, and the differential *KTN1* mRNA expression in the SNc between PD and controls, again suggest a functional role of *KTN1* in the development of PD.

PD is a complex disease that is affected by both genetic and environmental factors. For such a disease, both minor and major alleles of associated gene variants may represent the risk alleles (Kido et al., 2018). This may be explained by genetic drift, through which a slightly deleterious allele may have expanded in frequency and become a major allele (Ohta, 1987). Alternatively, a neutral or advantageous allele that was previously common may become a risk allele for PD owing to changes in the environment (Kido et al., 2018). In addition, overdominance, frequency-dependent selection, and gene–gene or gene–environment interactions may drive a major allele to become a risk allele for PD (Klitz et al., 1986). As observed in the current study, the major alleles G of rs8017172, T of rs17253792, and C of rs945270 in the H2 block, the three among the only four known GMV-associated alleles at *KTN1* (Hibar et al., 2015; Chen et al., 2017; Xu et al., 2017), significantly increased risk for PD, *KTN1* mRNA expression levels in the putamen, and the putamen GMVs. Further, we found increases, albeit not significant, in *KTN1* mRNA expression levels in the putamen in PD as compared to controls in one cohort. Together with the literature, these findings overall suggest a consistent, replicable, robust and positive relationship among the *KTN1* variants, *KTN1* expression in the putamen, putamen GMVs, and PD risk. The findings support the hypothesis that some risk *KTN1* alleles may increase kinectin 1 expression in the putamen, altering putamen GMVs and cognitive motor functions supported by the putamen, and lead to the clinical manifestations of PD.

It is well recognized that the dopaminergic neurons in the SNc are lost and the dopamine in the putamen is depleted in PD patients, which leads to a significant decrease in putamen (Schulz et al., 1999; Ghaemi et al., 2002; Krabbe et al., 2005; Pitcher et al., 2012; Sako et al., 2014) and SNc (Krabbe et al., 2005; Ogisu et al., 2013; Hikishima et al., 2015) volumes. As a compensatory response to loss of dopaminergic neurons and dopamine depletion (Hulshoff Pol et al., 2000; Krabbe et al., 2005), the remaining gray matter in the nigrostriatal pathway may compensate to maintain neural transmission by driving the expression of GMV-controlling proteins. One potential molecular mechanism involves the kinectin 1, as would be evidenced by higher levels of *KTN1* mRNA expression in the putamen and SNc in PD patients. *KTN1* has been reported to play a critical regulatory role in determining putamen GMV (Hibar et al., 2015; Xu et al., 2017; Luo et al., 2020b) (but not in SNc GMV yet). Kinectin 1 facilitates vesicle binding to kinesin, regulating crucial developmental processes including axonal guidance, vesicular transport of molecules, and apoptosis (Kumar et al., 1995; Hibar et al., 2015; Luo et al., 2020b), as well as neuronal cell shape and neuronal migration through kinectin–kinesin interactions (Zhang et al., 2010). Neurons with more kinectin 1 have larger cell bodies (Toyoshima and Sheetz, 1996; Zhang et al., 2010; Luo et al., 2020b), and thus may increase the putamen and SNc GMVs in subjects without brain disorders. Notably, the compensatory mechanism did not appear to restore the GMVs in subjects with PD, who have suffered loss of dopaminergic neurons in the SNc and dopamine depletion in the putamen. Alternatively, the increase of *KTN1* mRNA expression in the putamen and SNc in PD patients may reflect the consequences of long-term treatments with L-DOPA, or processes in relation to elevated α-synuclein, glycoprotein GPNMB, SN free water, iron accumulation, as has been observed in PD (Barbosa et al., 2015; An et al., 2018; Guttuso et al., 2018; Longhena et al., 2018; Moloney et al., 2018). This important issue warrants further investigation.

*KTN1* variants have also been associated with several other neuropsychiatric or neurodegenerative diseases/phenotypes before, including attention-deficit/hyperactivity disorder (ADHD) (Xu et al., 2017; Luo et al., 2020b), substance use disorder (SUD) (Li et al., 2016; Stringer et al., 2016; Luo et al., 2020a), and cognitive dysfunction in the elderly (Han et al., 2017). We noticed that slightly different sets of SNPs were associated with different disorders/phenotypes, which reflected the difference among them in genetic basis. However, we also noticed that some risk SNPs were shared between them, which reflected the commonness among them in some underlying mechanisms. In particular, the three functional SNPs (rs8017172, rs17253792, and rs945270) that most significantly regulated the putamen GMVs and *KTN1* mRNA expression in putamen were shared by PD and SUD, which were associated with reduced and enlarged putamen GMV, respectively. Substance may stimulate the dopamine release from enlarged putamen supported by elevated kinectin expression; however, in PD patients, reduced dopaminergic neurotransmission in the shrunk putamen may also drive kinectin expression via a compensatory mechanism, as discussed above and for ADHD (Luo et al., 2020b). This was why both PD and SUD were associated with GMV-associated alleles and elevated *KTN1* mRNA expression.

A major limitation of the present study is that the primary analyses focused on the cross-sectional or retrospective associations among *KTN1* SNPs, *KTN1* mRNA expression in the putamen and SNc, GMVs of putamen and PD. The findings revealed a statistical but not causal relationship. Future research to reveal the cause-effect relationships would require a prospective, functional study with an appropriate intervention, to answer whether *KTN1* variants regulate the development of putamen GMVs and PD, and whether the putamen GMV alteration results in or from the development of PD. The second limitation is that these associations were not analyzed in the same samples. For example, the SNP-mRNA and SNP-GMV associations were separately analyzed only in the samples without PD; and the SNP-PD association was analyzed only in the samples without GMV data; and therefore, we were unable to know the interactive impacts of SNPs, mRNA expression, putamen GMV and PD on one another from these separate samples. Future research to know their interactive impacts would require studying them in the same sample. Finally, some associations among lncRNAs, enhancers, *KTN1* mRNA, kinectin, and PD have never been studied, forming the research gaps as shown in Figure 1. Filling these gaps would be one of the future research directions.

In summary, the robust associations between *KTN1* variants and PD, the replicable associations between *KTN1* variants and putamen GMVs, the potential regulatory effects of *KTN1* variants on the mRNA expression in putamen and SNc and the activities of lncRNA and enhancer, the abundant *KTN1* mRNA expression in putamen and SNc, the differential mRNA expression in SNc between PD patients and controls, and the reported associations between putamen GMVs and PD, suggested that *KTN1* variants may underlie the putamen GMV and risk for PD.

## Data Availability Statement

The datasets generated for this study can be found in the dbGaP: Accession phs000196.v3.p1, phs000126.v2.p1, and phs001172.v1.p2.

## Ethics Statement

The studies involving human participants were reviewed and approved by the Human Investigation Committee of Yale University. The patients/participants provided their written informed consent to participate in this study.

## Author Contributions

XGL provided the resources (patients and data). QM, LF, XW, YZ, Y-CW, JJ, JX, HZ, CZ, KW, C-SL, and XGL conducted the study and performed the analysis. C-SL and XGL were responsible for the overall content as guarantors. All authors contributed to the formulation of overarching research goals and aims and the writing, reviewing, and editing of the article.

## Conflict of Interest

The authors declare that the research was conducted in the absence of any commercial or financial relationships that could be construed as a potential conflict of interest.

## Footnotes

1 http://enigma.ini.usc.edu/
